# Evaluation of epidemiological, clinical, and microbiological features of definite infective endocarditis

**DOI:** 10.3205/dgkh000286

**Published:** 2017-01-16

**Authors:** Reza Faraji, Mostafa Behjati-Ardakani, Seyed Mohammad Moshtaghioun, Seyed Mehdi Kalantar, Seyedeh Mahdieh Namayandeh, Mohammadhossien Soltani, Hengameh Zandi, Ali Dehghani Firoozabadi, Neda Tavakkoli Banizi, Foroozandeh Qasemi Kahtooie, Mehdi Banaei, Mohammadtaghi Sarebanhassanabadi

**Affiliations:** 1 Yazd Cardiovascular Research Center, Shahid Sadoughi University of Medical Sciences, Yazd, Iran; 2 Department of Biology, Faculty of Science, Yazd University, Yazd, Iran; 3 Cytogenetic Reproductive and Genetic Research Unit and Clinical Centre for Infertility, Shahid Sadoughi University of Medical Sciences, Yazd, Iran; 4 Department of Microbiology, Faculty of Medicine, Shahid Sadoughi University of Medical Sciences, Yazd, Iran

**Keywords:** infective endocarditis, heart valves, microorganisms

## Abstract

**Background:** Infective endocarditis (IE) is a microbial infection of heart valves and its endothelial lining which is considered as a life-threatening disorder. This study evaluated the epidemiological, clinical, and microbiological features of IE at the Cardiovascular Research Center in Yazd, Iran.

**Methods:** The cross-sectional study was conducted on 20 patients diagnosed with definite IE on the basis of Duke’s criteria hospitalized for one year in the Cardiovascular Research Center in Yazd, Iran, from January 2015 to December 2015. Demographic information, clinical, laboratory, and microbiological findings, and also trans-esophageal echocardiography (TEE) of each patient were recorded and assessed. The collected data were analyzed using SPSS 16.

**Results:** The mean age of the patients under study was 45±16 years with most of the afflicted patients (60%) being male. Most cases (70%) of IE were observed in the warm seasons (spring and summer). The most common clinical sign (80%) was fever. TEE was positive for all (100%) patients, and vegetation was seen in all patients. The nosocomial mortality rate was zero. However, 14 (70%) patients underwent surgical treatment. The valves afflicted with IE were: the mitral valve (40%), the aortic valve (35%), and the tricuspid valve (25%), respectively. 4 patients (20%) had a positive history of IE. Blood culture test was positive only in 1 case and the isolated microorganism belonged to the viridans group streptococci.

**Conclusion:** Despite the one-year high prevalence of IE in this study, the nosocomial mortality rate was not high and was reported to be nil under surgical and antimicrobial therapy.

## Zusammenfassung

**Hintergrund:** Die infektiöse Endokarditis (IE) ist eine mikrobielle Infektion der Herzklappen und ihrer endothelialen Auskleidung, die als lebensbedrohliche Erkrankung anzusehen ist. In dieser Studie wurden die epidemiologischen, klinischen und mikrobiologischen Merkmale der definitiven IE im Herz-Kreislauf-Forschungszentrum in Yazd, Iran untersucht.

**Methode:** Die Querschnittsstudie umfasste 20 Patienten mit definitiver IE, basierend auf den Kriterien nach Duke, die am Herz-Kreislauf-Forschungszentrum in Yazd, Iran, von Januar bis Dezember 2015 behandelt wurden. Demographische Angaben, klinische, Labor- und mikrobiologische Befunde sowie transösophageale Echokardiographie (TEE) jedes Patienten wurden beurteilt. Die gesammelten Daten wurden mit SPSS 16 analysiert.

**Ergebnis:** Das mittlere Alter der Patienten betrug 45±16 Jahre, wobei überwiegend (60%) männliche Patienten betroffen waren. Die meisten Fälle (70%) von IE wurden in den warmen Jahreszeiten (Frühling und Sommer) beobachtet. Das häufigste klinische Zeichen (80%) war Fieber. Die TEE war positiv für alle Patienten, wobei bei allen Patienten Vegetation beobachtet wurde. Die nosokomiale Sterblichkeitsrate betrug null. Allerdings bedurften 14 (70%) Patienten einer zusätzlichen chirurgischen Behandlung. Folgende Herzklappen waren am häufigsten betroffen: Mitralklappe (40%), Aortenklappe (35%) und Trikuspidalklappe (25%). Vier Patienten (20%) hatten eine positive Anamnese einer IE. Blutkulturen waren nur in einem Fall positiv; dabei wurden Viridans-Streptokokken isoliert.

**Schlussfolgerung:** Trotz der hohen Prävalenz von IE war die nosokomiale Sterblichkeit gering. Das ist jedoch nur durch entsprechende chirurgische und antimikrobielle Therapie erzielbar.

## Introduction

Infective endocarditis (IE) is a fatal disease. The term IE indicates the infection of the surface of cardiac endothelium suggesting the physical presence of microorganisms in the lesion. It is a serious and life-threatening condition, which may lead to death if untreated. Today, despite the medical and technological advancements in the field of pharmaceutical therapies of microbial infections and advanced surgical procedures, the mortality rate related to IE has not decreased [1], [2]. This condition is more prevalent in intravenous addicts and cardiovascular patients, specifically heart valve replacement patients [3] and congenital defect patients [4]. The symptoms manifest themselves as fever and chill, thoracic pain, arthralgia, dyspnea, lethargy, weight loss, diaphoresis, seizure, headache, splenomegaly, and cardiac failure [5]. The diagnosis of this disorder is established via Duke’s criteria including clinical, laboratory, and echocardiographic findings which diagnostically classify the IE into three categories: definite, probable, and rejected endocarditis. The presence of two major criteria or one major criterion plus three minor criteria or the presence of five minor criteria confirms the definite endocarditis. Also, the presence of one major criterion plus one minor criterion or the presence of three minor criteria makes endocarditis probable or plausible [6]. The most important laboratory finding for diagnosing IE is the culture of blood samples. Blood culture is important as it can determine the type of organism involved in the etiology of the disorder. However, in 10–25% of cases the result of this test is negative because of previous consumption of antibiotics [7], [8] or the presence of slow-growing or non-cultivable microorganisms [9]. The microorganisms most often involved in IE are *Staphylococcus aureus*, *Streptococcus pyogenes*, *Streptococcus viridans* group, and *Enterococcus* spp. [2], [10]. Sporadic cases of incidence of this disorder by other microorganisms have been also reported [11], [12], [13], [14], [15], [16]. The rate of incidence of IE has increased round the globe considerably. This is because of both, increased life expectancy and increased prevalence of addiction, especially intravenous drug abuse [10]. The average incidence of IE during 1993–2003 was 3.6/100,000 population in the community and the mean rate of nosocomial mortality was estimated to be 16% [7], [17]. The mortality rate associated with IE was 100% before the discovery of antibiotics; consequently, there was a significant decrease in the mortality rate after their discovery [5].

In the developed countries, the epidemiologic factors involved in the incidence of IE have changed compared to the past decades due to parameters such as increased life span, highly increased cases of nosocomial infections [18], [19], increased cases of degenerative valvular sclerosis, and mitral valve prolapse [19]. Nonetheless, there is little information available on this condition in the developing countries [20]. Moreover, there has not been sufficient research on the epidemiology of IE in Iran yet. Hence, the present researchers decided to assess the clinical, epidemiological, and microbiological findings on IE among the in-patients in the Cardiovascular Research Center in Yazd, Iran.

## Methods

### Patient population

This cross-sectional study was conducted during one year from January 2015 to December 2015 on in-patients hospitalized in the Cardiovascular Research Center in Yazd, Iran. This study was approved by the Committee of Ethics in Medical Research at the Shahid Sadoughi University of Medical Sciences, Yazd, Iran. Many patients, even non-aboriginal people, present to this center because of its advanced equipment and facility. Patients included into this study were all inhabitants from Yazd and especially from the eastern regions of Iran, such as Zahedan, Bandarabbas, and one patient from Afghanistan. The inclusion criterion was definite diagnosis of IE. The exclusion criterion was a final diagnosis of a disease other than IE. All included patients were diagnosed with IE based on Duke’s criteria. On this basis, the definite diagnosis was established in the case of the presence of two major criteria, one major criterion plus three minor criteria, or the presence of five minor criteria [6]. 

### Clinical and laboratory findings

The patients’ demographic information included age, gender, drug abuse, a positive history of cardiovascular disease, a history of cardiovascular surgery or artificial valve placement, a history of previous endocarditis, congenital cardiac disease, presence of bacteremia (including primary infections such as oral and dental infection, brucellosis), history of previous antibiotic consumption (and previous hospitalization in another hospital before presenting to our center).

The clinical findings included the following signs and symptoms at the time of admission: fever and chill, nocturnal diaphoresis, anorexia and weight loss, cardiac suffer, petechiae on body, ulcer nodes, arthralgia, and renal failure. Also, the laboratory findings included: leukocyte count, C-reactive protein (CRP), erythrocyte sedimentation rate (ESR), and microscopic hematuria. Moreover, the microbiological findings included all blood culture tests performed to assess the definite IE and to identify the microorganism involved in the etiology of IE (blood culture tests were completed using the Bactec laboratory method). 

### Echocardiography

TEE, when clinically indicated, was performed as described previously [18]. The investigators assessed the presence of following features: new dehiscence, vegetation, abscess, and new moderate or severe valvular regurgitation. An irregularly shaped echogenic mass, which was attached to a valve or myocardial surface, was considered as vegetation. The vegetation lengths were measured in different planes, and the maximal length was chosen. Also, a thickened area or mass with a heterogeneous echogenic or echoluscent appearance was considered as an abscess. Semi-quantitative analysis was performed to determine the acuity of the valvular regurgitation using color flow Doppler echocardiography.

### Statistical analysis

The required data were validated and imported into a database using Microsoft Access 2000. Statistical analysis was carried out using SPSS 16 for Windows and Microsoft Excel 2000. 

## Results

The findings obtained from 20 patients diagnosed with definite IE on the basis of Duke’s criteria showed that the mean age of the patients was 45±16 years. The youngest patient was a 2-year-old boy and the oldest patient a 76-year-old gentleman. Male patients were more frequently (60%) affected with IE than females. The ratio of males to females was 3 to 2. The disorder was observed more frequent (70%) in the warm seasons (spring and summer). The most common clinical sign was fever with a rate of 80% followed by chills with 30% and dyspnea with 25%. Four patients (20%) had a previous history of IE.

**Figure 1:**
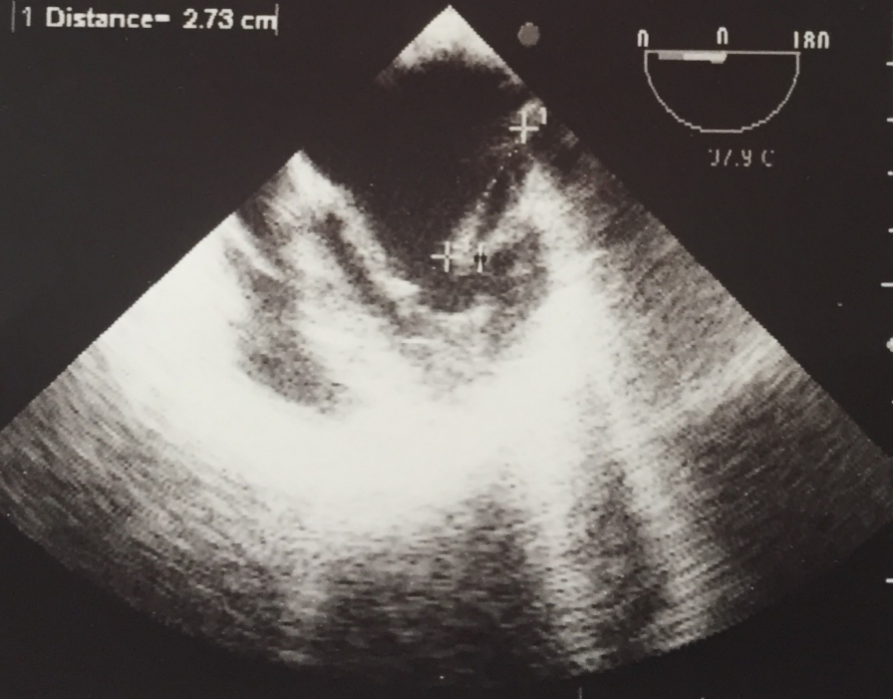
Vegetation in TEE

The nosocomial mortality rate was zero (Table 1 (Tab. 1)). However, 14 patients (70%) underwent surgical treatment. Four patients (20%) underwent valve defect repair and 10 patients (71%) underwent the valve replacement. Blood cultures were positive in only 1 patient, which yielded *Streptococcus viridans* group. The most common underlying disease (95%) was cardiovascular disease. Only one 2-year-old boy (5%) had an additional ventricular septal defect (VSD). Positive CRP was seen in 3 (15%) patients. 5 patients (25%) had an erythrocyte sedimentation rate greater than 20 mm in the first hour. Only one patient (5%) showed leukocytosis. Moreover, TEE was positive for all (100%) patients, and vegetation was seen in all. The vegetation size was large in 1 patient, i.e., almost 3 cm (Figure 1 (Fig. 1)). The most frequently involved valve was the mitral (bicuspid) valve (40%) followed by aortic valve (35%) and tricuspid valve (25%; Table 2 (Tab. 2)). All patients had a positive history of previous antibiotic administration and hospitalization in other hospitals before presenting to our center. The most frequently administered antibiotic was vancomycin in 70% of cases. Eighteen patients (90%) were discharged after hospitalization in good health condition. while 2 patients (10%) suffered from temperature at 38ºC at discharge.

## Discussion

IE is a fatal disease which is precipitated by predisposing factors such as underlying diseases, e.g., cardiac rheumatism and congenital cardiovascular defects. In recent years, the incidence of this disorder has increased due to heart surgeries, intravenous catheters, and drug abuse [5]. This study also reported the high prevalence (20 cases) of IE in one year. The mean age of the patients under study was 45 years. Similar to our study, in the study by Nunes et al. conducted on 62 patients in Brazil during 2001–2008, the mean age of the patients was 45 years [18]. Another study published by Al-Tawfiq et al. in Saudi Arabia during 1995–2008 reported a mean age of IE patients to be 59.7 years [21]. Moreover, the mean age of the IE patients was 57 years in a study conducted by Tornos et al. [19]. Nonetheless, contrary to our study, the mean ages of the IE patients in the studies conducted in India [22] and Tunisia [23] were 27.6 and 34.2 years, respectively. The results of our study and those of similar investigations with respect to the mean age of IE patients showed that cardiac rheumatism and untreated congenital heart defects are more prevalent among the younger individuals and in moderate-to-low income communities globally [22], [24].

However, the epidemiologic factors contributing to IE have changed in the developed countries over the past decades. The mean age of the IE patients in these countries was about 35 years before the discovery of antibiotics while the age of manifestation of this disorder has increased to 50 years in recent decades [1], [19]. Indeed, 50% cases of endocarditis have been reported in Europe and the USA to occur at the age of 60 years [1], [25], [26]. This change may be attributed to various factors such as increased life span, increased cases of sclerotic valve conditions in the elderly, significant decrease in the incidence of rheumatic fever, increased number of endocarditis patients following artificial valve replacement or intracardiac synthetic devices, increasing use of central vein catheter (CVP), and increased hospital-acquired (nosocomial) endocarditis [19], [27]. In our study males were more frequently (60%) affected by IE than females. In a study by Tornos et al. conducted in Europe, the ratio of male patients with IE was also greater than that of females [19]. In a study carried out in South America, men were more frequently (63%) afflicted with IE than women [18]. Furthermore, in the studies carried out in India, Pakistan, and Saudi Arabia, the rate of affliction with IE was higher in men compared to women [21], [22], [24]. In our study, fever was the most frequent clinical sign in patients, which is similar to other studies [13], [18], [23], [28], [29], [30], [31]. Also, in spite of the high prevalence of vegetation among the patients, the clinical signs were not seen in all patients indicating that clinical signs can not be the key to the essential diagnosis of cardiac involvement and therefore, radiologic studies like echocardiography are necessary for its determination. In our study, similar to a study conducted by Pereira et al., TEE indicated vegetation in all patients [29] as it was observed in our 20 patients. The mitral valve was most frequently (40%) involved followed by the aortic valve (35%) and tricuspid valve (25%). Murdoch et al. reported the most frequently involved valve (41.1%) to be the mitral valve followed by aortic valve (37.6%) [1]. Other studies, too, reported that the mitral and aortic valves were most frequently involved [21], [32]. Nevertheless, unlike our study, a study published by Netzer et al. reported that the aortic, mitral, and tricuspid valves were involved by 39%, 21%, and 2%, respectively [33]. Moreover, in the study of Pereira et al., the tricuspid valve (25%) and the mitral valve (25%) showed the highest involvement [29]. Regarding these findings, it could be concluded that TEE as a sensitive method can play a significant role in diagnosis of IE [34], [35]. In our study blood culture was positive in only 1 case (5%). The microorganism isolated form this case was a *Streptococcus viridans* group. All our patients had a positive history of previous antibiotic administration. Negative blood culture is reported in many studies [6], [24], [36], [37], [38], [39]. The spectrum of microorganisms is also different. Yet, the microorganism most frequently involved in the etiology of endocarditis is reported to be *Staphylococcus aureus* [1], [18], [21], [32], [33], [34], [35], [40], [41], [42], [43]. Endocarditis with negative blood culture occurs in 2.5–31% of cases [34] mostly due to initiation of antibiotic therapy before culture, infection with highly fastidious bacteria, or non-bacterial pathogens [7], [37], [39], [44]. These negative blood cultures may be detrimental to patients as they delay the diagnosis of endocarditis exerting a negative consequence on treatment [45]. Factors such as the culturing method, blood volume, number of blood cultures, and meticulous care at blood sampling affect the results of blood culture greatly [18].

In our study, the rate of nosocomial mortality was zero, which is less than the reported elsewhere at 8–15% [31], [32]. Also, positive CRP was seen in 15% of the patients. Additionally, 5% of the patients had leukocytosis and 25% of the patients had ESR >25%. RF was not positive in any of the patients. Our study is limited by the relatively small sample including patients from a single large tertiary-care center. Many of these patients with IE were referred from other hospitals. Acknowledging the small number of the patients in our study, it seems mandatory to design and implement future studies in which factors like leukocytosis, high CRP, high ESR, and hematuria as minor criteria are considered with an assessment of a greater number of IE cases.

## Conclusion

Regarding the negative results of cultures due to the previous administration of antibiotics and its comparison with TEE, it could be concluded that TEE is a more reliable method for diagnosing IE compared to the culturing method.

## Notes

### Competing interests

The authors declare that they have no competing interests.

### Acknowledgements

The authors of this article give their special thanks to Yazd Cardiovascular Research Center and Shahid Sadoughi University of Medical Sciences, Yazd, Iran.

## Figures and Tables

**Table 1:**
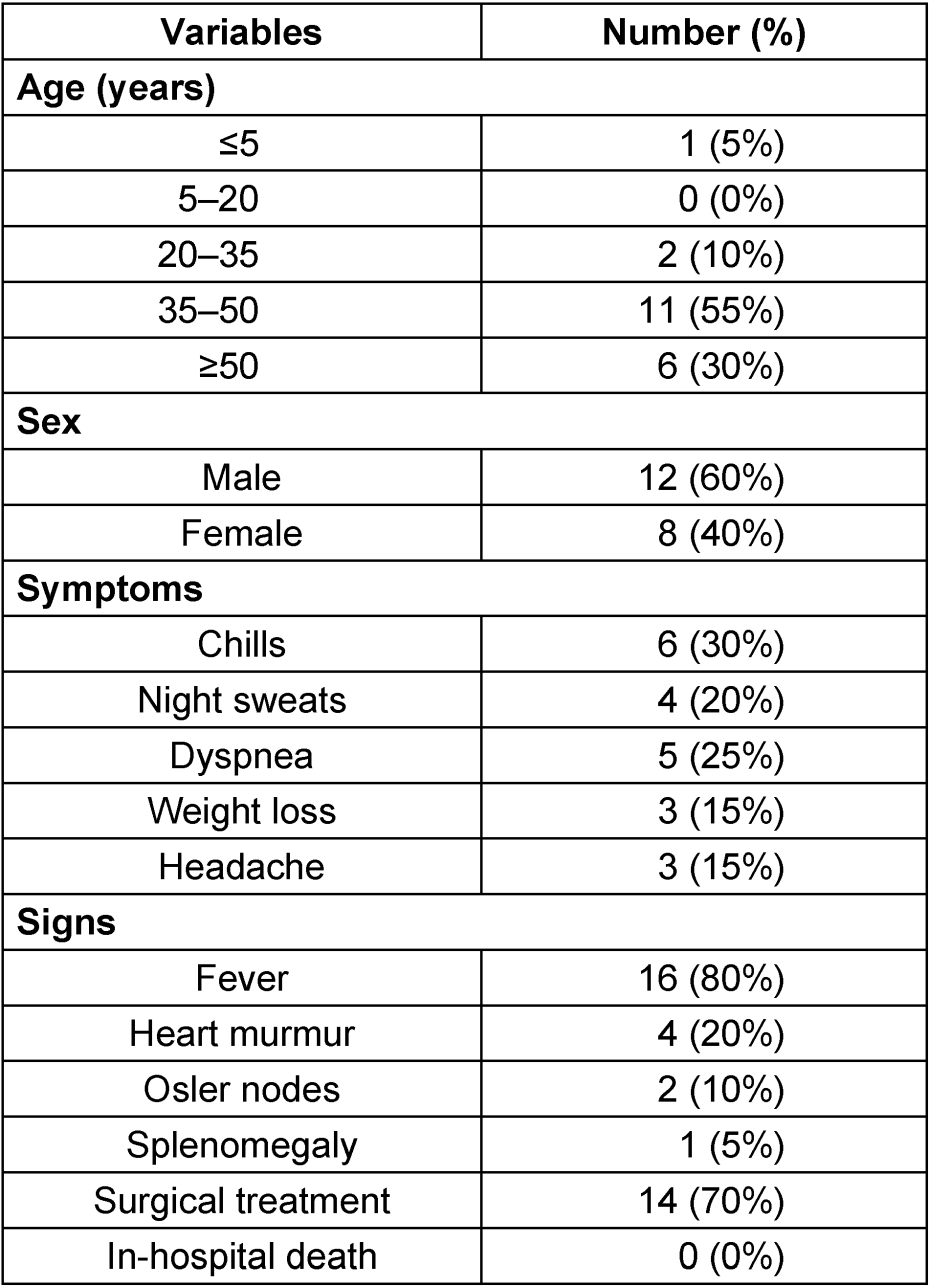
Demographic and clinical information of patients with IE

**Table 2:**
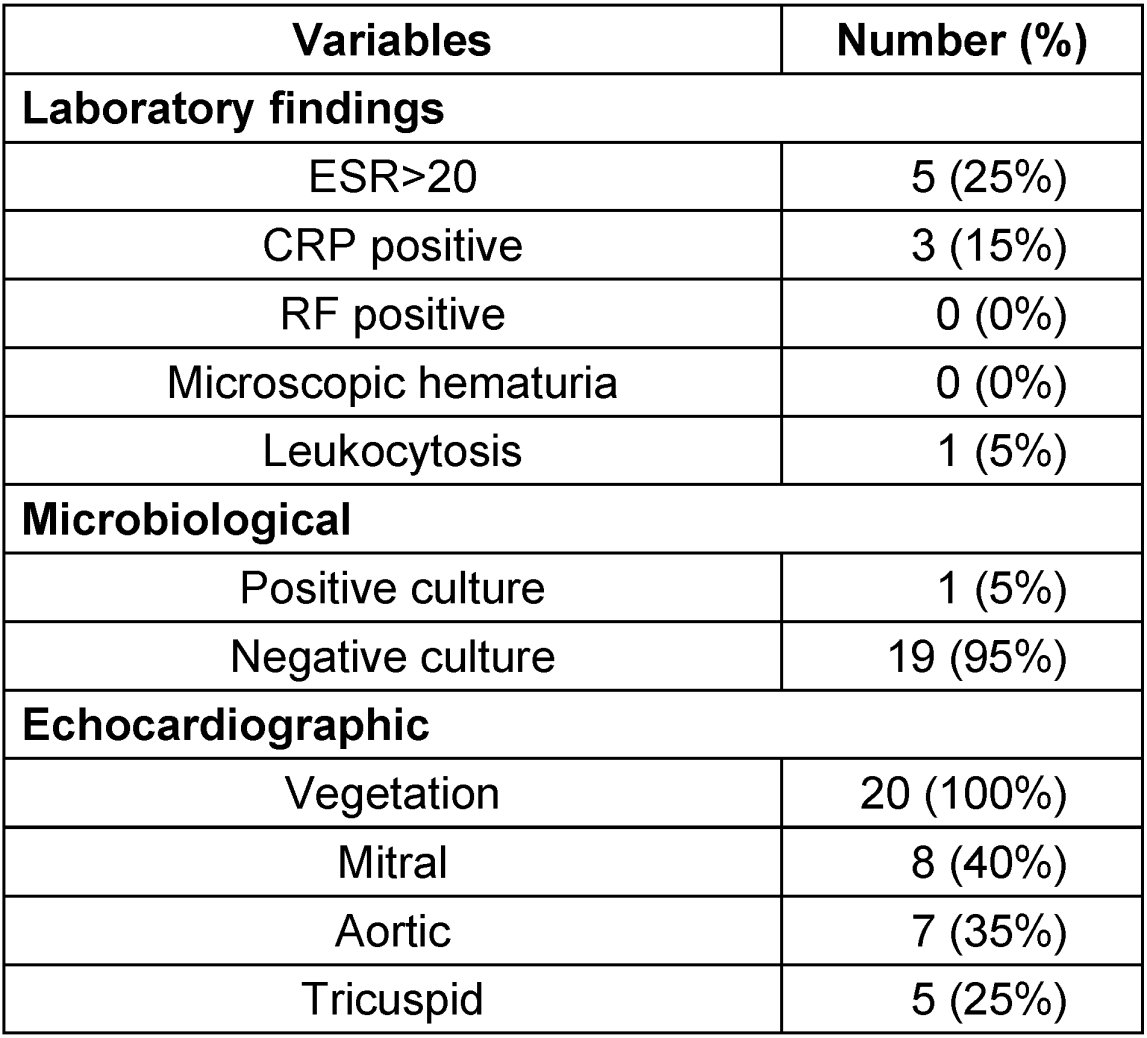
Results of laboratory and TEE features of patients with IE
